# The Use of Omic Technologies Applied to Traditional Chinese Medicine Research

**DOI:** 10.1155/2017/6359730

**Published:** 2017-01-31

**Authors:** Dalinda Isabel Sánchez-Vidaña, Rahim Rajwani, Man-Sau Wong

**Affiliations:** ^1^Department of Rehabilitation Sciences, The Hong Kong Polytechnic University, Hung Hom, Kowloon, Hong Kong; ^2^Department of Health Technology and Informatics, The Hong Kong Polytechnic University, Hung Hom, Kowloon, Hong Kong; ^3^Department of Applied Biology and Chemical Technology, The Hong Kong Polytechnic University, Hung Hom, Kowloon, Hong Kong

## Abstract

Natural products represent one of the most important reservoirs of structural and chemical diversity for the generation of leads in the drug development process. A growing number of researchers have shown interest in the development of drugs based on Chinese herbs. In this review, the use and potential of omic technologies as powerful tools in the modernization of traditional Chinese medicine are discussed. The analytical combination from each omic approach is crucial for understanding the working mechanisms of cells, tissues, organs, and organisms as well as the mechanisms of disease. Gradually, omic approaches have been introduced in every stage of the drug development process to generate high-quality Chinese medicine-based drugs. Finally, the future picture of the use of omic technologies is a promising tool and arena for further improvement in the modernization of traditional Chinese medicine.

## 1. Natural Products and Traditional Chinese Medicine in Drug Discovery

Since ancient times, plants have been an essential element for the prevention and treatment of a wide variety of diseases. Historically, natural products represent one of the most important reservoirs of structural and chemical diversity for the generation of leads in the drug development process [1, 2]. The tendency to develop drugs from natural sources can be clearly observed in a study of the sources of drugs between 1981 and 2010. The study showed that about 45% of the approved drugs by the FDA were natural products or natural products derivatives [3, 4].

In the past years, the use of herbal preparations has gained attention in European and Asian countries. Only in Europe, about 100 million people make use of traditional and complementary medicine. Furthermore, the increasing popularity of traditional and alternative medicine is observed in Africa, Asia, Australia, and North America [5–7]. In academia and industry, a growing number of researchers have shown interest in the development of drugs based on Chinese herbs [2].

**Figure 1:**
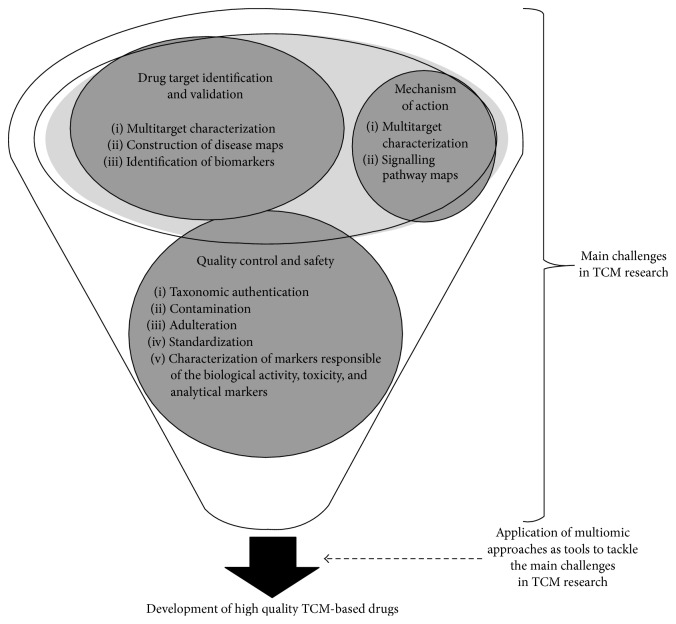
Application of omic technologies to tackle the main challenges in TCM research.

Traditional Chinese Medicine (TCM) is a medical system for the prevention and treatment of diseases that focuses on the patient rather than the disease when compared to the Western medicine. The main principle by which TCM works is the use of herbs for the restoration of the yin-yang imbalance that results in disease [2]. Despite the increasing popularity and interest on TCM, researchers face a challenging task when gathering scientific evidence and clinical validation of Chinese based herbal remedies. The main bottlenecks in the study of TCM include quality control, the identification of cellular targets, mechanism of action, and clinical validation due to the variability of the individual herbal ingredients, the complexity of herbal formulations, and the combined action on different targets (Figure 1) [6, 8].

Novel advanced technologies are needed to improve separation methods, quality control, standardization techniques, screening, the study of the mechanism of action of individual compounds, and clinical validation assays. In this sense, the application of omic technologies in TCM research is a promising approach to assist in the modernization of TCM and to address the complex challenges encountered in TCM research. Therefore, the aim of this review is to give a general overview of the use of omic technologies as promising and powerful tools in TCM research.

## 2. Omic Approaches in TCM Research

The rapidly evolving technology has led to the development of research tools to assist a more comprehensive study of biological systems. In TCM, researchers have gradually introduced the most recent technological advances trying to overcome the most common bottlenecks in TCM research. By analyzing the emergence and evolution of the current technologies, their potential application in TCM research can be better understood.

A significant breakthrough in technological advances was the completion of the Human Genome Project which is considered one of the greatest scientific achievements of the past century [9]. The genomic revolution in the Human Genome Project was the platform that contributed to the development and improvement of technologies for identification of drug targets, target validation, and disease etiology [10, 11]. Some of the technological advances include Sanger DNA sequencing, nanotechnology, miniaturization and automation technologies, DNA-based genetic markers, cloning systems, polymerase chain reaction, and genotyping of single nucleotide polymorphism. The techniques developed during the Human Genome Project have played an essential role in the understanding of biological processes [11].

Despite the significant contribution of genomic studies, the need to bridge the sequence information for the identification of potential therapeutic targets with the physiology and pathology of an organism using novel sophisticated approaches became a clear and pivotal task [10]. One way to fill the gap between genomic information and biological processes was the use of combined strategies from several levels together with analytical technologies and improved computational power. The integration of technology, bioinformatics, and molecular biology approaches at different organizational levels is comprised in systems biology and provides a complete picture to understand the molecular mechanisms [12, 13].

**Figure 2:**
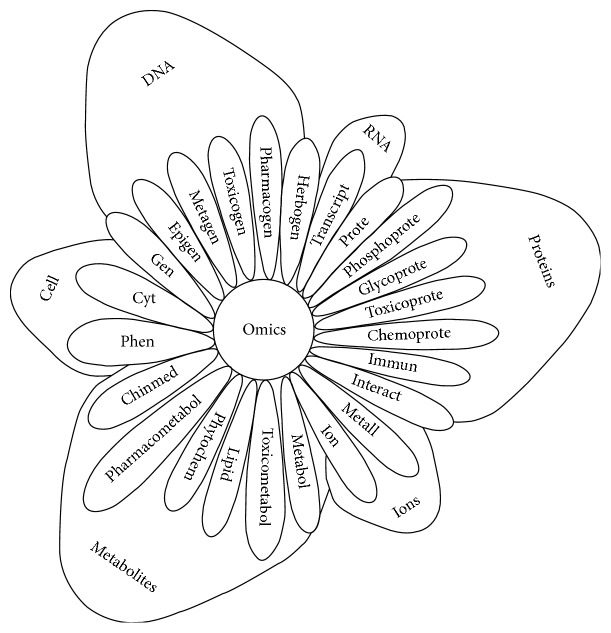
Omic approaches and their area of study in systems biology.

The postgenomic era was a pivotal stage for the emergence of “omic” studies in biological research representing the beginning of the systems biology era. Data generated by omic studies are described as the wholeness of living systems that result in useful information after the application of bioinformatic analyses [14]. The organizational levels in systems biology include genomics, transcriptomics, proteomics, and metabolomics [12]. Furthermore, new omic concepts and technologies have been introduced comprising more specialized areas of study in systems biology [14] (Figure 2).

The analytical combination of information from each omic approach is crucial for understanding the working mechanisms of cells, tissues, organs, and organisms as well as the mechanisms of disease [11, 15]. Visualization and analysis of combined multiomic data increase, in a large scale, the comprehensive understanding of biological processes [14]. Therefore, application of omic approaches in TCM represents powerful tools for the development of high-quality herb-derived drugs.

### 2.1. Omic Approaches at DNA Level

Genes and gene products act as a complex interconnected network in synchrony with a wide variety molecular system pathways rather than acting independently. Since the establishment of omics approaches, omic technologies have been applied in different areas of biotechnology, drug discovery, and biomedical research including TCM. However, a close analysis of the omic research published in the past ten years shows that the use of omic technologies in TCM has not been fully explored [16]. Therefore, special attention should be paid to link the advantages of omic techniques with the complex concerns encountered in TCM research.

Another omic approach at DNA level is epigenomics. Epigenetic studies focus on the reversible heritable modifications in gene expression that do not involve any change in the DNA sequence [14, 17]. As an omic approach, the aim of epigenomics is to identify gene alterations through the analysis of epigenetic changes that take place in the whole genome [14]. Despite promising findings, the application of epigenomics in TCM needs further exploration [17].

Metagenomic studies involve the analysis of a collection of genes from the environment that are related to the host physiology [14]. This approach is of particular interest when studying the relationship between the gut microflora and the pathophysiological state of the host and drug metabolism [18]. So far, the contribution of metagenomics in TCM research represents a novel and promising area of study that contributes to the modernization of TCM.

The study of the alterations in gene expression upon exposure to chemicals to identify potential genetic toxicants and monitor their cellular mechanisms is the main focus of toxicogenomics [14, 19]. Toxicogenomics comprises the dynamics between genes and environmental stress in disease etiology and development [20].

Pharmacogenomics aims to identify and analyze genes involved in the response to drugs to assess interindividual variability and susceptibility to adverse drug reactions [21, 22]. Pharmacogenomics studies provide valuable information to determine doses for the effective and safe use of medications [14]. In the field of TCM research, databases have been constructed including active compounds found in Chinese herbs which are categorized using chemical informatics protocols. Multidisciplinary integration of data generated by other omic approaches provides a wider and more understanding overview of pharmacogenomics applied to TCM research.

Herbogenomics, also referred as botanogenomics, is a novel concept that makes use of genomic and proteomic comparative analysis for the study of the biological effect of herbs [23]. This concept has emerged as an attempt to introduce a more specialized platform particularly focused on herbal remedies [23, 24]. The comparison between treated and control groups gives an insight into the genome and proteome alterations that take place upon treatment with herb-derived compounds or herbal preparations.

### 2.2. Omic Approach at RNA Level

Transcriptomics comprises the study of the complete set of messenger RNA under specific conditions in any given cell; it is also known as the study of transcripts [12, 25]. By using genetic association databases, it is possible to make comparisons between the gene expression profile under treatment with certain herbal formulae and the profile of disease state or any known drug. Such comparisons can be used to predict unknown physiological effects, unwanted interactions, or side effects [16]. Nonetheless, more transcriptomic information combined with multiomic technologies should be included in strategies for modernization of TCM.

### 2.3. Omic Approaches at Protein Level

The above-mentioned omic approaches have contributed to the understanding of biological processes for the evaluation and validation of TCM. Another promising technology developed that has strongly emerged as a powerful tool for the assessment of physiological events is proteomics. Since the introduction of the term proteomics for the first time 20 years ago, proteomic technologies have rapidly evolved and being integrated into several stages in the pharmaceutical research and development [26]. Proteomic studies focus on the whole population of proteins in a specific cell type, tissue, body fluid, or a whole organism at a given time [14, 27]. The concept of proteomics can be described from the point of view of TCM in which the Qi or vital essence is the result of the overall performance of the proteome [28].

The downstream complexity of mechanisms that lead to a specific biological effect is not directly related to the number of genes. Instead, other gene expression mechanisms such as posttranslational modifications and alternative splicing play an important role in protein variation. It has been estimated that about 35% of human genes undergo alternative splicing which means that the traditional one gene one protein hypothesis should be adjusted to a one gene-many protein model [13, 25]. As a consequence, proteomics has a particularly important impact on the discovery of novel drug targets [26].

Phosphorylation is considerably the most commonly found posttranslational modification in proteins accounting for about 17,500 phosphorylated gene products in humans [29]. Phosphorylation of proteins plays a pivotal role in crucial cell mechanisms including gene expression, cell signaling, metabolism, cell growth, and cell differentiation [14]. In addition, alterations in protein phosphorylation have been directly involved in disease etiology and progression, but only a small number of proteins, about 350 proteins, have been experimentally validated [29]. Despite the enormous potential of phosphoproteomics technologies, the difficulties encountered in the development of sophisticated, sensitive, and reliable phosphoproteomic methods may contribute to the slow introduction of these techniques in TCM studies.

Similarly, as phosphoproteomics, glycoproteomics comprises the study of proteins with another important posttranslational modification in cell processes, namely, glycosylation. Glycosylation of proteins occurs in about 25% of the human proteins and nearly 4,500 proteins have being detected to undergo glycosylation [29]. However, some estimations reveal that glycosylation may occur in about 50% of all human proteins. Glycosylated proteins participate in cellular functions including cell adhesion, cell immunity, protein translation, and protein degradation among other cellular functions [14]. Although glycoproteomic technologies are currently available and have shown great potential in drug discovery and drug development, the application of these technologies in the development of high-quality TCM-derived drugs needs to be explored.

Another omic approach that focuses on the proteome is toxicoproteomics which is used to evaluate the effect of the exposure to toxic agents in cells and tissues at qualitative and quantitative level. The proteome profiling upon xenobiotic exposure allows prediction of toxic responses to herbal components [30].

Chemoproteomics applies function-based proteomics to study the protein structure and their interaction with chemicals. The proteins subjected to chemoproteomic studies require carefully optimized conditions to conserve protein structure, posttranslational modifications, and even interactions with regulatory proteins [14, 31]. Chemoproteomic approaches can be categorized as global and targeted chemoproteomic strategies. The global chemoproteomics approach focuses on the cellular response, namely, protein expression or any specific posttranslational modification, upon treatment with a compound of interest [31]. At the end of the analytical process, bioinformatic tools are applied to characterize protein structure and function [14].

Immunomics focus on the regulatory mechanisms of the immune system on pathogens from antigen recognition to the immune response caused by the antigens presented to the immune machinery of the host [14, 32]. Although the immunomodulatory effects of several Chinese herbs are well known and documented, the use of modern technology to assess the close interactions and molecular mechanisms triggered by TCMs using immunomics is still in a very early stage [33].

As mentioned above, the use of multiple omic approaches allows a broader and more detailed understanding of biological phenomena from different points of view. The complexity and a large amount of data generated by each individual omic approach have led to the emergence of a new omic tool to combine, analyze, and interpret multiomic data. The result of the analytical combination of the enormous amount of data generated by different omic approaches is called integrome and it is the main focus on interactomics [34]. Interaction networks can be constructed using bioinformatic tools in interactomic studies to track changes in the traits of networks. The main application of interactomic studies so far has been in drug discovery [14].

### 2.4. Omic Approaches at Metabolite Level

Metabolomic studies include the qualitative and quantitative analysis of a large number of metabolites and their dynamics in a given biological system [12, 35]. The aim of metabolomics is the measurement of the global, dynamic, and downstream homeostatic effect in biochemical pathways caused by specific stimuli [16]. The stimuli that induce changes in the metabolic profile can be categorized as internal factors such as genetic modifications, as well as external factors such as environmental and pharmaceutically induced effects [12]. The metabolic profiling can also provide important information about the pathophysiological, pharmacodynamics, and pharmacokinetic condition [16]. Any change in the living system in terms of physiology, pathology, or any other disturbance will lead to changes in the metabolome [14].

The metabolic state of an individual is influenced by environmental, genetic, and gut microbiome factors. Comparative studies of the metabolic profiles before and after drug exposure provide valuable information about the treatment outcome and the effect of drugs on metabolism. Pharmacometabolomics is currently applied in pharmacology, drug discovery, and development and personalized therapy [36]. In recent years, pharmacometabolomic studies have been widely applied in TCM studies contributing to a better understanding of the metabolic profile-TCM effect.

The omic discipline that combines metabolomics and toxicology in comparative analyses of endogenous metabolite profiles in a normal state and during and after drug treatment is toxicometabolomics [37]. Since safety is one of the main concerns in TCM research, metabolomics and toxicometabolomics are very useful tools to assess the toxicological processes in TCMs [14]. For instance, several studies investigated the mechanistic basis of aristolochic acid nephrotoxicity using 1H-NMR spectroscopy, GC-MS, and LC-MS [38–41]. In this regard, toxicometabolomic approach uncovered that aristolochic induced nephrotoxicity in aristolochic acid treated rats may be arising from decreased levels of prostaglandins resulting in a reversible vasoconstrictive state and kidney lesions [38–41]. A similar investigation was also conducted to investigate the nephrotoxic effect of long-term usage of ricin-based products. Ricin is a water-soluble glycoprotein based key toxin of* Ricinus communis* (also known as castor-oil plant). Long-term treatment with ricin was shown to induce perturbations in multiple metabolic pathways including amino-acid metabolism and oxidative stress which partly explains the observed nephrotoxicity [42]. Furthermore, a GC-MS study investigated hepatotoxic effects of triptolide (the active anti-inflammatory ingredient of* Tripterygium wilfordii*) and discovered changes in ß-oxidation pathway [43]. Together, these toxicometabolomic studies demonstrated the potential of metabolomic technologies for understanding TCM-based toxicity.

Lipids play essential roles in biological processes from energy reservoirs and structural cellular components to elements in cell-signaling pathways [44]. Studies on the lipidome, the lipid profile in biological systems, have shown that disturbances in lipid metabolites are closely related to the development of diseases such as obesity, Alzheimer's disease, diabetes, and some infectious diseases [14, 45]. However, analytical challenges have been encountered in lipidomic studies due to the chemically diverse structure of lipids in complex biological mixtures. Therefore, the technologies used in lipidomic studies require constant improvement to provide more sensitive and accurate analysis of the lipid profile in biological samples.

The phytochemome comprises the group of compounds present in plants such as alkaloids, polyphenols, terpenes, sulfides, thiols, plant peptides, and their metabolites. Phytochemomics focuses on the intracellular and extracellular study at different molecular levels, the chemical structure, and mechanism of action of the phytochemome [46]. Phytochemomic analytical approaches use the same methods that are used in metabolomics studies. The main difference between metabolomics and phytochemomics is that the latter only focuses on the chemical analysis of compound present in plants. The phytochemomic concept has not been widely used. Therefore, it is more common to find in the literature the term metabolomics when studying phytochemicals.

The focus of chinmedomics is to investigate the biological action, synergistic effect, and metabolic profile of Chinese formulae. Chinmedomics has shown great potential when studying TCM since it introduces the holistic concept of TCM combined with the most sophisticated analytical tools [47, 48].

The technologically driven advances in chinmedomics have been used to address the efficacy and safety concerns in TCM research [47, 49]. To ensure the therapeutic effect of TCM treatment, the serum pharmacochemistry analysis is assessed to collect information about the mechanism of action, the interaction among the components of the formulae, and the assessment of the relationship between syndrome and TCM.

### 2.5. Omic Approaches at Ion Level

Metallomics is defined as the comprehensive quantitative and qualitative analysis of an entire set of metals and metalloid species within a cell, tissue, or the whole organisms [50]. Since it is well known that a lot of proteins require the presence of metals such as copper, ion, zinc, and molybdenum as cofactors to exert their biological actions, metallomics significantly contributes to studying protein function [14].

Crucial physiological processes such as cellular signaling, metabolism, enzymatic activity, and transmembrane transport involve the action of ions [14]. The ionome, a concept introduced 10 years ago that refers to the study of the mineral nutrients and trace elements in a cell or organism, is the focus of ionomic studies [51, 52]. The quantitative measurement of changes in the mineral composition of a specific tissue or organism as a response to internal or external stimuli, namely, normal or pathological physiological processes, drug effect, or genetic modifications, is carried out in ionomic studies [53].

### 2.6. Omic Approaches at Cellular Level

The phenotype is the whole set of physical and biochemical characteristics of a living organism as a result of interaction with genetic and environmental factors [14, 54]. In the disease state, the disease manifestation, progression, severity of the symptomatology, and response to therapy are closely related to the genetic component of the organism and all together defines the disease phenotype [54].

Phenome is the whole set of expressed traits in a defined population associated with the influence of genetic of environmental factors [14, 55]. Phenomics focuses on the understanding and monitoring of the changes in the phenome as a result of environmental and genetic factors [14]. By studying the phenotype, the biological processes that occur as a result of genetic and environmental influences can be reflected in the genomic architecture [55]. Phenomic studies are difficult to carry out regarding TCM since it is complex to identify the compounds responsible for the therapeutic effect due to the rich chemical content of herbal remedies. However, the integration, analysis, and understanding of phenomic data applied to TCM studies represent a valuable tool in TCM research.

The study of the phenotype at a cellular level for the understanding of molecular disease networks is the focus of cytomics. In order to gain more understanding of the molecular processes at a cellular level, the human cytome project was conceived [56]. However, the application of these technologies in TCM research is quite limited.

As discussed above, the diversity of omic approaches provides a wide variety of technological tools that are applied to TCM research. In addition, the potential applications of some omic technologies in TCM research should be further explored. In Table 1, a summary of all the omic technologies discussed above and their application in different areas of the drug discovery and drug development process in TCM research is presented.

Omic technologies can be applied to the study of a wide variety of diseases as shown in Table 2. Therefore, the potential use of omic technologies in TCM research has a wide scope of application.

## 3. Quality Control and Safety of TCM Using Omic Technologies

A critical concern in TCM research is to ensure quality, safety, and efficacy to meet the quality standard requirements [93]. In the regulatory context, the assessment of herbal medicinal products has been included in protocols and guidelines developed by regulatory authorities and monitoring agencies such as the European Medicines Agency, the European Food Safety Authority, the Chinese State Food and Drug Administration, the Herbal Medicinal Product Committee (European scientific committee), and the World Health Organization. The latest has published a series of test methods for identification, purity, and content assessment in the quality control Methods for Medicinal plant materials [94–97]. The European Medicines Agency has developed guidelines including the guideline on good agricultural and collection practice for starting materials of herbal origin, guideline on quality of herbal medicinal products/traditional herbal medicinal products, guideline on the quality of combination herbal medicinal products/traditional herbal products, and the guideline on specifications: test procedures and acceptance criteria for herbal substances, herbal preparations, and herbal medicinal products/traditional herbal medicinal products [98]. In order to meet the criteria established by local and international regulatory agencies for quality assurance, safety, and efficacy in traditional medicine, omic technologies have played a crucial role in facilitating the sensitive and efficient evaluation of herbal-based medicines.

In TCM, the diversity and number of components responsible for the biological activity make the analytical procedure a challenging task. However, emerging technologies and improved methods have contributed to facilitating the quality control of TCMs. Some of the technologies applied in the characterization of metabolites for quality control include NMR-based metabolic fingerprinting, capillary electrophoresis, HPLC, gas chromatography, HPLC-PDA-ESI/MS, HPLC-DAD-ESI/MS, GC/MS, GCxGC-qMS, TOF/MS, GCxGC-TOF/MS, MALDI-TOF/MS, HLPC/UV, TLC, ultra-TLC, UPLC, UPLC-Q/TOF-MS, IT-TOF-MS, capillary zone electrophoresis (CZE), capillary electrochromatography (CEC), pressurized CEC, micellar electrokinetic chromatography, turbulent flow chromatography (TFC), and HPLC/ESI/MS [93, 99–101]. Some of the above-mentioned technologies including MALDI-TOF-MS and UPLC-Q/TPG/MS have been adapted to high-throughput settings to support a simple, quick, sensitive, and cost-effective analysis of TCMs [80, 100, 102]. More recently, technology such as miniaturized and integrated microchips, that is, as Herbochip®, DNA chip, protein chip, and DNA chip, have been developed to be applied in high-throughput screening of active ingredients [93]. Therefore, DNA barcoding technologies have also shown to facilitate the standardization, identification, and quality control of herb-based medicines [103]. 1H NMR spectroscopy is an analytical tool that allows the detection of proton-bearing compounds without making any distinction based on the chemical compound class. This technique is used to identify and quantify metabolites in a mixture in a one-step process which facilitates the analysis in high-throughput settings [104, 105] and is a powerful tool in the quality control of herbal remedies. For example, 1H NMR was used to determine the metabolic profile of the Chinese formula Danggui Buxue Tang traditionally used in the treatment of menopausal symptoms. The analysis of the 1H NMR spectra of the herbal formula revealed a distinctive metabolic profile of the decoction when compared to the individual components of the formula which provides pivotal information for the standardization and quality control of the herbal remedy [105]. UPLC-MS/MS is being widely employed for the analysis of samples of complex composition since it provides high resolution for the detection of compounds present in herbal mixtures without the need for pure standards [106].

The metabolite characterization is not only useful for assessment of efficacy and safety by determination of the compounds responsible for the biological activity or side effects, but also it is essential for the standardization during the manufacturing process. Differences in quantity and quality of compounds in TCMs can be due to factors such as the plant of the plant used, storage conditions, agricultural practices (growth conditions, geographic area, and time of harvest, among others), extraction, and preparation methods [15, 35]. Those factors can contribute to the variability in content and quantity of compounds between batches that could affect the pharmacological activity [107]. Therefore, metabolome characterization using the previously mentioned techniques is essential to ensure the development of high-quality TCMs.

Although important technological advances for the analysis of TCMs have been developed and applied, some challenges are still of significant concern. Frequently, some of the components responsible for the therapeutic or toxic effect in TCM have not been fully characterized which hampers the analytical process [102, 108]. Another limitation in quality control of herbal preparations is the lack of standards for the assessment of markers. However, alternative analytical methods such as quantitative analysis of multicomponent by a single marker (QAMS) have shown to be effective for the simultaneous analysis of multiple compounds [102].

In conclusion, the use of omic technologies has enormously contributed to improving the cost effectiveness, sensitivity, and reliability of analytical methods in the quality control process of TCM. Several analytical techniques are being introduced in quality control of herbal preparations at all stages of the production process. The need to have one optimized analytical procedure for one specific herbal preparation slows down the development of analytical protocols for TCM when compared to the analytical procedures of Western medicine. More extensive and oriented work is needed to overcome the issues encountered and to adopt the newest technological advances in TCM research. However, the introduction of existing technologies and the fast development of emerging omic tools provide a wide variety of analytical platforms and promising future to assist in the quality control of TCM.

## 4. Omic Technologies in the Study of Potential Therapeutic Targets

The study of therapeutic targets in TCM is a complex and challenging task. It requires the use of multiple methodologies combined to construct disease maps from different perspectives since the effect of TCMs is the final result of the effect on multiple targets. From the genomic point of view, about 30–40 thousand genes encode a larger number of proteins that represent potential drug targets for human diseases [14]. In this arena, omic technologies provide a wide variety of tools for the study of disease mechanisms and potential therapeutic targets.

As mentioned above, genomic technologies were the first methods to be applied in target discovery in combination with transcriptomics. Microarray analysis and mRNA expression profiles used to compare the normal state, disease related genes, or gene expression under treatment conditions have shown promising results for target validation. However, the gene expression profiles might not be directly correlated with the late functional biological state [14]. Therefore, limitations of genomic studies in target discovery and disease mechanisms should be addressed by combining the information collected with the use of other omic approaches such as proteomics.

The use of proteomic and metabolomic approaches applied to TCM research has increased since 2006. On the other hand, genomics and transcriptomics have not been widely used in TCM research when compared to proteomics and metabolomics as shown in observations made until 2012. Therefore, there is a clear need to explore genomic and transcriptomic technologies applied to TCM research [109].

Proteomic studies have contributed to the identification of potential drug targets not only by comparing the proteome profiles but also by using protein-drug or protein-protein interaction information [14]. In vitro and in vivo comparative proteomic studies using TCMs allow the identification of proteins that are present in the disease state when compared to the proteome of the normal state and those proteins can be considered as potential drug targets. After protein identification, the potential targets should be properly validated to ensure their involvement in the therapeutic effect of the compound or group of compounds studied. In TCM, identification of drug targets is essential for approval of TCM base drugs [110].

A close analysis of the methods used in proteomics for the study of the mechanism of disease has highlighted the pros and cons of such methods. For instance, a limitation being pointed out is the use of gel-based proteomics. Gel-based separation techniques can lead to partial peptide recovery from the gel which requires the use of several extraction steps to increase the peptide yield. However, the multistep methodologies are time-consuming compared to gel-free proteomic techniques. It has also been observed that the resolution of 1D gel systems is not good enough to analyze complex biological samples [111]. Furthermore, 2D gels have been a key method in comparative proteomic since their introduction in the mid-70s [61]. 2D gel technologies have partially overcome the problems encountered in the use of 1D gels; however, the poor resolution when high abundant proteins are present in the sample represents another issue in gel-based methods. In addition, gel-based technologies are time-consuming compared to other separation methods such as chromatographic techniques. In order to overcome the main problems observed in gel-based separation techniques, gel-free proteomics coupled with MS have shown to be a more efficient option in proteomic studies [111].

Proteomic studies can be combined with virtual screening technologies to predict binding profiles of the TCM components with potential target proteins and to build protein-protein interaction maps to track the signaling pathways involved in the biological effects. Furthermore, the combined data from comparative proteomics, protein-drug, and protein-protein interaction provide useful information to support the process for candidate drug targets [14]. Important advances in MS techniques have been a key element in the study of the molecular targets of natural products [112]. Particularly, MS techniques namely MALDI-MS and ESI-MS/MS have been widely applied in target discovery studies. In natural product research, MS methods coupled to separation techniques such as affinity chromatography, including small molecule-immobilized chromatography, biotin-streptavidin affinity chromatography, and photoaffinity labeling, have shown to be a powerful combination for target identification [112].

The study of posttranslational modifications (PTMs) in proteomics has enormously contributed to the understanding of molecular mechanisms in biological systems particularly for the identification of potential drug targets. The study of PTMs in biological systems is a challenging task due to the high diversity of PTMs. About 300 different types of PTMs have been identified using the most sophisticated analytical technologies [113]. New analytical platforms for the study of PTMs include PTM peptides enrichment steps after protein extraction and digestion that are followed by multidimensional analyses such as LC-MS/MS [113, 114]. Chromatographic-based methods are also being used in PTM studies including hydrophilic interaction chromatography (HILIC), chemical derivatization methods, enzymatic labeling approaches, and immunoaffinity chromatography methods for the study of glycopeptides [113]. Other chromatographic techniques used include the metal oxide affinity chromatography (MOAC) and immobilized metal ion affinity chromatography (IMAC) which are the most popular methods for the study of phosphoproteins [113, 115]. Other types of PTMs such as ubiquitination are studied using antibodies for enrichment purposes combined with chromatographic techniques coupled with MS. MS/MS technologies combined with enrichment methods, such as acetyl-lysine antibodies, are used for the analysis of protein acetylation [113, 114]. Finally, protein methylation can be assessed by LC-MS/MS, SILAC-MS combined with enrichment techniques such as ion exchange chromatography, HILIC, and isoelectric focusing [113]. The information obtained from the study of PTMs using MS technology has contributed to the understanding of the signaling networks involved in disease development and drug activity representing one of the key approaches to generate information at a protein level for drug development purposes. Therefore, it is essential to apply the most advanced methods developed in TCM to contribute to a more efficient, sensitive, and time effective evaluation of TCMs.

Nowadays, proteomics technologies comprise a wide variety of high-throughput methods to study complex systems. A large amount of proteomic data generated can be analyzed using open source or commercial bioinformatics. Nevertheless, new bioanalytical approaches need to be developed to manage the enormous amount of proteomic data to improve the handling, analysis, and visualization of proteome information [62]. In TCM research, the use of the most recent analytical tools is growing slowly. On the other hand, the evolution of the omic technologies and the history of the implementation of omic technologies in TCM have shown that great efforts are being made to introduce those technologies in TCM research.

The comparison of the abnormalities in the metabolome to the normal stage provides an insight into the pathways affected at several disease stages, and it is known as comparative metabolomics [116]. For instance, in vitro, in vivo, and clinical studies have been carried out using MS and NMR technologies for the assessment of the metabolome in tumor diagnosis and evaluation of prognosis and to investigate the effect of the treatment [116]. Comparative omic approaches are useful tools that provide information about the disease state to identify the genes/proteins/metabolites involved in disease development compared to normal states [14]. A general overview of the application of omic technologies in differential or comparative studies indicated that coronary heart disease, chronic liver disease, hypertension, chronic kidney disease, hyperlipidemia and atherosclerosis, chronic stomach disease, and diabetes mellitus are the most common disorders studied in clinical TCM using omic technologies [109].

Furthermore, metabolomic studies have been shown to be helpful in the detection of response-to-treatment biomarkers which can be used for the development of new drugs [116]. Metabolic and inflammatory disorders are good examples in which metabolome profiling can assist in the identification of potential drug targets and disease biomarkers [14, 116]. The study of disease biomarkers provides information about diagnosis and disease development [117]. Recently, a proteomic technology that has been used in the identification of disease biomarkers is UPLC-MS^E^. This approach involves the use of parallel alternating scans to generate ion information using low collision energy or to generate a full scan mass fragment, precursor ion, and neutral ion information by high collision energy. The information obtained by MS^E^ has been shown to provide similar data to the data obtained by conventional MS/MS analysis. However, UPLC-MS^E^ technology has the advantage of allowing the characterization of unknown biomarkers in an untargeted context [118]. For example, UPLC-MS^E^ was used to study the metabonomic profile of chronic kidney disease induced by adenine excess and the protective effects of the TCM* Poria cocos* [119]. This metabonomic approach allowed the identification of disease biomarkers that can be also used to explain the mechanism of action of* Poria cocos* [119]. In other studies, the protective effect of rhubarb and ergone against chronic kidney disease was evaluated using UPLC-QTOF/HDMS which provided valuable information about the pathogenesis of chronic kidney disease, identification of bioactive fractions, and the mechanism of action of both herbal mixtures [120, 121]. Another analytical tool is GC-MS which is considered a basic analytical tool in metabolomic studies together with NMR and LC-MS [122]. An example of the application of GC-MS in TCM research is a study in which a metabolomic profiling of depressed patients treated with Xiaoyaosan, a Chinese formula used for the treatment of depression and schizophrenia, was determined [122]. The metabolic profile obtained by GC-MS in this study allowed the monitoring of changes in the metabolome present in the urine of patients receiving Xiaoyasan treatment which provided more information about its regulatory effect of neurotransmitter metabolism and its beneficial effects on depression [122].

In high-throughput settings, metabolomics technologies are used not only for the identification of biomarkers but also for understanding the progression and the metabolome variation in the different disease stages which contributed to a better understanding of the pathology picture [116]. In addition, metabolomic profiling is a useful tool for the diagnosis and study of the pathological mechanisms. Nowadays, metabolomics approaches are being explored and introduced in the development of TCMs [14].

Methods such as NMR-based metabolite profiling, HPLC, GC-MS, GCxGC-TOF-MS, MALDI-TOF-MS, and CE-MS are metabolomic tools that can be used for the simultaneous analysis of 1000 compounds. Such technologies have shown to be fast, efficient, and sensitive for the detection of low abundance metabolites. In terms of bioinformatics analysis, great efforts are being made to improve the current tools in order to integrate the omic information and generate more reliable and complete bioinformatics tools. An example of such improved bioinformatic options is the Integrated Interactome System which comprises genes, proteins, metabolites, and drugs data [62]. Together with other bioinformatics tools such as the Human Metabolome Database, BioMagResBank, the Madison Consortium Database, MassBank, and the Golm Metabolome Database, metabolomics studies significantly contribute to picture detailed understanding of disease mechanisms and the identification of drug targets [116].

Metabolomics profiling can assist in the identification of early biomarkers of disease which is beneficial to detect early pathological stages [123]. Early disease biomarker detection is crucial for the implementation of prevention and therapeutic schemes in clinics. Therefore, sensitive detection methods to be developed and applied in TCM research are highly demanded. Great advances in the application of metabolomics techniques are taking place and the application of the most novel methods in TCM is being carried out. In addition to the omic data generated in TCM studies, pharmacological evaluation for target validation purposes in in vitro and in vivo models are needed to complement the information obtained by omic studies [14].

## 5. Studying the Mechanism of Action of TCMs Using Omic Approaches

Chinese formulations comprise a combination of several herbs. The active compounds in the formulations can act on specific targets and the biological activity is the result of combined effects. The final pharmacological action of the active compounds can be mutual accentuation, mutual enhancement, mutual suppression, mutual antagonism, or mutual incompatibility [6]. In order to develop drugs based on Chinese medicine, the mechanism of action and the identification of other cellular targets should be properly established to validate the pharmacological effect and determine possible side effects [110]. However, the multitarget actions of a large number of ingredients present in the formula represent a daunting resource-intensive challenge for TCM research, especially because most of the cellular targets and the mechanisms of action are unknown [6, 16, 112, 124].

There is an overlap in the methodologies for identification of disease mechanisms, biomarkers, drug targets, and the study of the mechanism of action of TCMs. Some of the technologies mentioned in the previous section are also used in the study of the molecular mechanisms in TCMs. Therefore, the most recent advances in omic technologies that have being applied in TCM research including some practical examples and technologies that need further exploration will be discussed.

Genomic approaches to the study of the mechanism of action of TCMs have been applied in the past years. A clear example of the use of recent sophisticated genomic technologies is the biclustering analysis of gene expression profiles using DNA microarray technology to identify gene expression variation. The data generated in the DNA microarray analysis is used to identify biologically relevant clusters for gene expression profiles resulting from chemical treatment to construct connectivity map (cMap) profiles. Consequently, the cMAP profiles are processed by biclustering analysis using a factor analysis for bicluster acquisition method. The data generated by the above-mentioned tools combining gene expression data, statistics, and bioinformatics contributed to the elucidation of the mechanism of action of TCMs and have shown potential for the analysis of complex herbal formulae [125].

A combination of technologies such as isoelectric focusing, SDS-PAGE, in-gel digestion, MALDI-TOF-MS, and real-time PCR have contributed to the identification of proteins involved in the mechanism of action of curcumin in in vitro models. Additionally, information about the signaling pathway in which the differential proteins act can provide with information about the function and signaling pathway involved in the mechanism of action [126]. In another study, a proteomic analysis using 2D gels, in-gel digestion, and MALDI/TOF/TOF coupled with bioinformatic analysis to obtain the protein-protein interaction network showed the identification of proteins and signaling pathways involved in the effect of curcumin on gastric cancer cells [127]. The studies mentioned above highlight the enormous amount of information and understanding of the mechanistic actions behind the effect of naturally derived compounds which supports the potential of omic studies in TCM research.

Another detailed study of the use of proteomics to determine the mechanism of action of TCMs was carried out by Lao et al., 2014. In this review, the authors summarize a number of studies based on proteomics in TCM research carried out in the past 10 years. In this review, it can be observed that proteomic technologies are being applied in the study of a wide variety of disorders in in vitro, in vivo, and in silico studies [128]. Proteomic technologies such as 2D gel electrophoresis, MALDI-TOF-MS combined peptide mass fingerprint, and the use of Mascot software are used to elucidate the mechanism of action of triterpenes in leukemia cells. The study showed the successful identification of proteins associated with the antitumor mechanism of TCM [129]. The mechanisms of action involved in the hepatoprotective effects of a TCM were investigated using a combination of 2D gel electrophoresis, MALDI-TOF/TOF-MS, and the construction of protein-protein interaction networks using bioinformatics tools (STRING). Identification of 7 target proteins was possible suggesting the potential of the proteomic strategy for the elucidation of the mechanism of action of TCMs [130]. Another recent study has implemented iTRAQ-based quantitative technology for the identification of disease biomarkers and the mechanism of action of a Chinese medicine on a Parkinson's disease model. In this study, a detailed protein network map was constructed showing the molecular pathways involved in the neuroprotective effects of the medicinal plant studied and the identification of potential disease biomarkers [74].

When studying the mechanism of action of TCMs, the use of transcriptomic technologies applied to TCM is powerful tools due to the high sensitivity, accuracy, specificity, reproducibility, and high-throughput approach [131]. In addition, microarray technologies for the simultaneous monitoring of a large number of genes represent a very convenient platform for TCM studies. With the availability of well-established protocols for sample preparation, RNA isolation, and amplification, hybridization and data analysis have made this technology a valuable tool in TCM. Microarray technologies have been used to elucidate the mechanism of action of individual compounds or mixture of compounds in TCM studies. Taken together with databases of gene expression profiles of TCMs, construction of the drug-gene interaction signature provides information on the molecular pathways involved [131].

An example of the use of transcriptomics in the study of the mechanism of action of TCMs is the study carried out by Liu et al., 2013. In this study, differential microarray gene expression analysis in an in vitro platform combined with pharmacological studies for validation purposes to elucidate the mechanism of action of the TCM studied. The data generated were analyzed using the software ArrayTrack, CLUSTER, and TREEVIEW. The approach used improved the understanding of the mechanism of action and the potential use of the TCM in cancer treatment [89]. Additional examples of studies carried out on TCMs using omic approaches to elucidate the mechanism of action are shown in Table 3.

The use of metabolomics technologies provides a deeper insight into the metabolic changes accompanied by TCM treatment which enables biomarker identification and elucidation of mode-of-action. Danqi Tongmai tablet was shown to have a protective effect against acute myocardial infarction. Comparison of metabolomic profile of Danqi Tongmai tablet treated and untreated rats were subjected to pathway analysis to identify the pathways modified in Danqi Tongmai tablet treated rats. Briefly, the knowledge from Kyoto Encyclopedia of Genes and Genomes (KEGG) was utilized to identify deregulated pathways. A more detailed statistical test of pathway enrichment and comparison of pathway topology were conducted in MetaboAnalyst [79]. The study demonstrated a systems-level analysis of metabolic profile for understanding TCM effector mechanism.

Although omic technologies have being applied in the study of the molecular mechanism by which TCMs exhibit their biological effects, more exploration is needed to take full advantage of the wide range of technologies currently available.

## 6. Conclusions

Omic technologies have shown enormous potential in the modernization of TCM. Gradually, omic approaches have being introduced in every stage of the development process to generate high-quality TCMs. The complexity of TCM in terms of composition and multitarget effects has being addressed by omic approaches. However, further study and optimization of the current methodologies are needed to successfully overcome the most common bottlenecks in TCM research. The high chemical and pharmacological diversity found in TMCs requires the development of a suitable set of omic technologies established in a strategic manner and adapted to every specific compound, a group of compounds, or TCM extracts. Because each plant, formulae, extract, or group of compounds has different physicochemical nature and contains different active and toxic compounds as well as analytical and active biomarkers, each case requires specific analytical approaches and methodologies that cannot be applied to another plant, formulae, extract, or group of compounds. The wide variety of technologies available needs to be further explored and the feasibility of any particular omic technology or the combination of multiomic approaches needs to be studied in detail. Therefore, validation of the strategic omic approaches applied is needed to support their introduction in guidelines and to allow the development of standard operation procedures to facilitate reproducibility, harmonization, and standardization of the protocols used in the development of TCMs. Emerging novel and sophisticated omic technologies will require a continuous study and application in TCM research. Finally, the future picture of the use of omic technologies is a promising tool and arena for further improvement in the modernization of TCM.

## Figures and Tables

**Table 1:** Omic technologies and their areas of application in TCM research.

Omic approach	Area of application	Ref.
Genomics	Drug target identification Genetic disease markers Genome manipulation for target validation	[57–59]

Epigenomics	Study of epigenetic mechanisms of phytochemicals in the regulation of gene expression	[17]

Metagenomics	Study of the relationship between the gut microflora and the pathophysiological state of the host and drug metabolism	[18]

Toxicogenomics	Mechanism of toxicity (chronic, carcinogenic effect, and secondary effects of drugs) Biomarkers of toxicity and disease biomarkers Gene expression profiling of contaminants of herbal remedies for quality control Detection of toxic adulterants in herbal preparations Screening tests for prediction of toxicity of herbs in the drug development process	[14, 20, 21]

Pharmacogenomics	Candidate genes with potential relevance in the efficacy and safety profile of TCMs Drug targets Identification of active compounds linking gene and disease information	[60]

Herbonomics	Mechanism of action of herbal components Toxicity assessment of herbal remedies	[23, 24]

Transcriptomics	Prediction of therapeutic potential and safety	[16]

Proteomics	Therapeutic targets Exploration of pharmacological effect and mechanism of multitarget regulatory actions Quality control and toxicological profile of herbal remedies Disease diagnosis Biomarkers and monitoring of disease progression	[15, 16, 61, 62]

Phosphoproteomics	Study of disease pathogenesis Disease biomarkers Drug targets	[14, 63]

Glycoproteomics	Identification of aberrant proteins linked to pathophysiological processes Diagnosis biomarkers Mechanism of disease Drug targets	[14]

Toxicoproteomics	Detection of biomarkers in response to xenobiotic exposure Prediction of toxic response to herbal components	[30]

Chemoproteomics	Mechanism of disease Mechanism of action of compounds of interest	[14]

Immunomics	Drug target Biomarker identification Study of immunomodulatory effect of herbs	[14, 62]

Interactomics	Drug discovery Building interaction networks among interacting molecules	[14, 29]

Metabolomics	Pharmacokinetics Pharmacodynamics Toxicology Disease diagnosis Molecular pathology Botanic identification Standardization of herbal remedies Quality control Elucidation of toxicological profile of herbal preparations	[64]

Toxicometabolomics	Toxicity related biomarkers Molecular pathways involved in toxicological responses to botanicals	[14]

Lipidomics	Mechanism of disease Drug target discovery Disease biomarkers	[14, 33, 45]

Phytochemomics	Mechanism of action of herbal compounds Quality control of herbs Production of herb-based medicinal products Toxicity assessment	[34]

Pharmacometabolomics	Study of phenotype signatures by mapping key pathways of the metabolic drug effects Pharmacology Drug discovery and drug development Personalized medicine	[63]

Chinmedomics	Mechanism of action and metabolic profile of Chinese formulae Efficacy and safety of TCMs Disease biomarkers	[35–37]

Metallomics	Functional study of metal/metalloid-containing molecules and metabolites (i.e., genes, polysaccharides, and proteins) Mechanism of action of herbs Disease biomarkers Toxicological profile Quality control	[14, 45, 46, 65]

Ionomics	Mechanism of disease Mechanism of action Disease biomarkers Toxicological profile Quality control	[50, 51]

Phenomics	Disease biomarkers Biomarkers of survival Therapeutic targets	[66]

Cytomics	Disease biomarkers Drug targets Target validation Personalized medicine Drug development Pharmacology Toxicology	[14, 54, 67]

**Table 2:** Examples of application of omic approaches in TCM research per area of study in systems biology.

Level in system biology	Omic approach	Disease/health condition	Results	Ref.
DNA	Genomics	Cancer	The different signaling pathways affected by the compound Kushen injection, a Chinese formula used for the treatment of different types of cancer, that contribute to its antitumor activity were identified including cell proliferation and apoptosis	[68]
	Epigenomics	Cancer	Gene expression modulatory pathways were elucidated in cancer models of naturally occurring compounds such as curcumin isolated from turmeric, epigallocatechin-3-gallate which is a component of green tea, genistein isolated from soy, isothiocyanates present in broccoli, sprouts, and wasabi, lycopene which is found in tomatoes, and resveratrol present in grapes, wines, and eucalyptus	[17]
	Metagenomics	Gut microflora disturbances	The Chinese formula containing Radix ginseng, Rhizoma atractylodis macrocephalae, Poria and Radix glycyrrhizae traditionally used in the treatment of spleen deficiency showed restoring effects of the gut ecosystem by modulation of the gut microflora composition and structure of the intestinal mucosa	[18]

RNA	Transcriptomics	Liver diseases	Using microarray analysis, it was observed that the components of the three yellows heart-draining decoction (San-Huang-Xie-Xin-Tang) exert their liver protective activity by acting on the antiproliferation activity modulated by P53 and DNA damage signaling cascade	[69]

Protein	Proteomics	Leukemia	The Chinese medicine Patrinia heterophylla, used as an antitumor herb, affected the regulation of proteins involved in energy metabolism, oxidative stress, apoptosis, signal transduction, differential induction, and protein biosynthesis which give insights into the antitumor mechanism of action of this herb on leukemia K562 cells	[70]

Metabolite	Metabolomics	Diabetes type 2	Berberine, a compound isolated from the Chinese herb Coptis chinensis, showed to downregulate high levels of 13 free fatty acids in patients with diabetes type 2 and dyslipidemia. A better understanding of the signaling pathways involved in the mechanism of action of berberine including fatty acid, insulin resistance, and glucose pathways was elucidated using metabolomic analysis	[70]
	Toxicometabolomics	Nephrotoxicity induced by aristolochic acid (compound widely present in botanicals)	The mechanism of toxicity of aristolochic acid was elucidated and it involved direct cytotoxic effect and inhibition of the enzyme phospholipase A2 which renal function leading to renal failure	[39]
	Lipidomics	Hyperlipidemia	Poria cocos, a medicinal fungus used in TCM, induced positive changes in the fatty acid and sterol profile in an animal model for hyperlipidemia. Also, the signaling pathways affected by *Poria cocos* were identified using lipidomic analysis	[71]
	Phytochemomics	Cancer	The antioxidant capacity of 7S and its deglycosylated form D7S soy protein was evaluated. Both peptides showed antioxidant activity against free radicals and showed the effect on proliferation, oxidative status, and differentiation of H-Caco-2 cells	[72]
	Chinmedomics	Liver disorders	The Chinese formulae Liu Wei Di Huang Wan improved the restoration of the metabolic profile that was disturbed as a result of inflammation processes	[73]

**Table 3:** Studies on TCM using omic technologies (P, proteomics; T, transcriptomics; M, metabolomics; PG; pharmacogenomics; G, genomics; L, lipidomics).

Disease	TCM	Omic approach: method	Target or signaling pathway	Reference
Parkinson's disease	*Acanthopanax senticosus*	P: iTRAQ	Inhibition of the expression of Lewy bodies; modulation of mitochondrial energy metabolism, axonal transport, and protein degradation; suppression of endoplasmic reticulum stress and apoptosis; maintenance of centrosome integrity, iron, and calcium homeostasis	[74]

Anxiety	Radix Rehmanniae Preparata	P: 2DGE, MALDI-TOF/MS	Inhibition of MSG-induced downregulation of *β*-synuclein, DJ-1, peroxiredoxin-2, peroxiredoxin-6, DDAH-1, and iron-sulfur proteins	[75]

Cerebrovascular disease	Tao Hong Si Wu decoction (Semen Prunus, Flos Carthami, Radix Rehmanniae Preparata, Radix Angelicae Sinensis, Rhizoma Ligustici Chuanxiong, and Radix Paeoniae Rubra)	P: 2DGE, MALDI-TOF-MS	Regulation of Nrf2-mediated phase II enzymes	[76]

Chronic renal injury	Fu-Ling-Pi (*Poria cocos*)	M: UPLC Q-TOF/HSMS/MS^E^	Regulation of adenine and amino acid metabolism	[77]

Cardiovascular and kidney disease, cancer	Venenum bufonis	M: NMR	Cardiac acute toxicity by inhibition of the Na+/K+ -ATPase pump, activation of the mitochondrial apoptotic pathway, increasing the levels of ROS, disturbance of mitochondrial function, and induction of apoptosis	[78]

Acute myocardial ischemia	Danqi Tongmai tablet (Salvia miltiorrhiza and Panax notoginseng)	M: LC-LTQ-Orbitrap MS	Regulation of the tricarboxylic acid cycle, amino, and nucleotide metabolism	[79]

Atherosclerosis	Xin-Ke-Shu (Salvia miltiorrhiza, Pueraria lobate, Panax notoginseng, Crataegus pinnatifida, Aucklandia lappa)	M: UPLC-Q-TOF MS	Regulation of fatty acid, beta-oxidation pathway, sphingolipid metabolism, glycerophospholipid metabolism and bile acid biosynthesis, proteolysis, citrate cycle, lysine and glutathione, glycerophospholipid, taurine, hypotaurine, tryptophan, and arachidonic acid	[80, 81]

Hemolytic and aplastic anemia	Gui-Xiong (Angelicae Sinensis Radix and Chuanxiong Rhizoma)	M: UHPLC–Q-TOF/MS	Regulation of thiamine and sphingolipid metabolism	[82]

Rheumatoid arthritis	Huang-Lian-Jie-Du-Tang	M: LC–Q-TOF-MS	Restoration to normal urinary levels of citric acid, creatine, pantothenic acid, carnitine, pantothenic acid, phenylacetylglycine and plasma levels of uric acid, L-histidine, and L-phenylalanine	[83]

Systemic lupus erythematosus	Jieduquyuziyin (*Radix Rehmanniae, Carapax Trionycis, Herba Artemisiae Annuae, Rhizoma Cimicifugae foetidae, Herba Hedyotidis, Radix Paeoniae Rubra, Herba Centellae Asiaticae, Semen Coicis, Fructus Citri Sarcodactylis and Radix Glycyrrhizae*)	M: RRLC-Q-TOF/MS	Regulation of unsaturated fatty acids and phospholipid metabolic pathways	[84]

Gastric cancer	*Salvia miltiorrhiza*	T and P: next generation sequencing-based transcriptomics and iTRAQ	Inhibition of the metabolism of glucose in gastric cancer cells and cell growth	[85]

Cancer	*Cantharis vesicatoria*	PG: PCR array, microarray-based transcriptome-wide mRNA expressions, and COMPARE analysis	Induction of apoptosis by regulation of tumor suppressors p53, p21, mitochondrial Bax and Bcl-2 proteins, JAK/STAT pathway, NF-KB, and oxidative stress that leads to DNA damage	[86]

Colon, ovarian, and lung cancer	*Artemisia annua*	PG: microarray-based transcriptome-wide expression profiling and compare analysis	Regulation of TNF, tumor suppressor p53, c-Myc, and Max-mediated transcriptional control of gene expression	[87]

Ischemic stroke	Bu-yang-Huan-wu decoction (*Astragalus membranaceus, Angelica sinensis, Peonia lactiflora, Ligusticum chanxiong, Prunus persica, Carthamus tinctorius* and *Pheretima aspergillum*)	G: affymetrix gene chip array	Downregulation of apoptosis, inflammation, angiogenesis, and blood coagulation genes; upregulation of neurogenesis and nervous system development genes	[88]

Breast cancer	Si-Wu-Tang extract (*Paeoniae, Angelicae, Chianxiong *and* Rehmanniae*)	T and G: microarray transcriptional profiling	Upregulation of GREB1, EGR3, PGR, and inhibition of cell growth.	[89]

Lipopolysaccharide induced diseases	Pulsatillae decoction (*Radix Pulsatillae, Rhixoma Coptidis, Cortex Phallodendri and Cortex Fraxini*)	G: affymetrix chip	Reduction of lipopolysaccharide-induced damage, improvement of the physiological and biochemical responses to overcome the action of lipopolysaccharides	[90]

Dyslipidemia	Review on different TCMs	Several approaches used such as G, T, and M	Review on different TCMs	[91]

Cancer	*Panax ginseng*	Several approaches used such as G, P, and PG	Stimulation of angiogenesis, modulation of PI3k and AKT pathways, regulation of cell adhesion, migration, and cytoskeleton. Effect on nuclear steroid hormone receptors	[92]
